# Metagenomic Analysis: Alterations of Soil Microbial Community and Function due to the Disturbance of Collecting *Cordyceps sinensis*

**DOI:** 10.3390/ijms252010961

**Published:** 2024-10-11

**Authors:** Yangyang Chen, Zhenjiang Chen, Xiuzhang Li, Kamran Malik, Chunjie Li

**Affiliations:** 1 State Key Laboratory of Grassland Agro-Ecosystems, Key Laboratory of Grassland Livestock Industry Innovation, Ministry of Agriculture and Rural Affairs, Engineering Research Center of Grassland Industry, Ministry of Education, Gansu Tech Innovation Centre of Western China Grassland Industry, Centre for Grassland Microbiome, College of Pastoral Agriculture Science and Technology, Lanzhou University, No. 222, Tianshui South Road, Chengguan District, Lanzhou 730020, China; chenyy2023@lzu.edu.cn (Y.C.); malik@lzu.edu.cn (K.M.); 2 Qinghai Academy of Animal and Veterinary Science, Qinghai University, Xining 810016, China; xiuzhang11@163.com

**Keywords:** *Cordyceps sinensis*, Chinese *Cordyceps*, soil microorganisms, metagenomic, microbial community, microbial function

## Abstract

Soil microorganisms are critical to the occurrence of *Cordyceps sinensis* (Chinese *Cordyceps*), a medicinal fungi used in Traditional Chinese Medicine. The over-collection of Chinese *Cordyceps* has caused vegetation degradation and impacted the sustainable occurrence of *Cordyceps*. The effects of Chinese *Cordyceps* collection on soil microorganisms have not been reported. Metagenomic analysis was performed on the soil of collecting and non-collecting areas of production and non-production areas, respectively. *C*. *sinensis* collection showed no alteration in alpha-diversity but significantly affected beta-diversity and the community composition of soil microorganisms. In *Cordyceps* production, Thaumarchaeota and Crenarchaeota were identified as the dominant archaeal phyla. DNA repair, flagellar assembly, propionate metabolism, and sulfur metabolism were affected in archaea, reducing the tolerance of archaea in extreme habitats. Proteobacteria, Actinobacteria, Acidobacteria, Verrucomicrobia, and Nitrospirae were identified as the dominant bacterial phyla. The collection of Chinese *Cordyceps* enhanced the bacterial biosynthesis of secondary metabolites and suppressed ribosome and carbon metabolism pathways in bacteria. A more complex microbial community relationship network in the Chinese *Cordyceps* production area was found. The changes in the microbial community structure were closely related to C, N, P and enzyme activities. This study clarified soil microbial community composition and function in the *Cordyceps* production area and established that collection clearly affects the microbial community function by altering microbial community structure. Therefore, it would be important to balance the relationship between cordyceps production and microbiology.

## 1. Introduction

Grasslands continuously supply abundant biological resources and ecological services for mankind [1]. They also significantly contribute to nutrient storage (C, N and P), biogeochemical cycling [2,3] and climate regulation [4]. However, grasslands are characterized by dynamic changes [5] and are susceptible to climatic factors and human activities [6]. Although climate change is a driver of grassland degradation, the degree and extent of its impacts are often enhanced or weakened by human activities [7,8]. For ecologically fragile grassland ecosystems on the Tibetan Plateau, human activities can cause severe ecosystems damage and grassland degradation [9].

Soil microorganisms are essential for maintaining the stability of soil ecosystems [10]. Microorganisms are involved in the cycling of key nutrients (C, N, and P) and break down the carbon compounds that make up soil organic matter (SOM) [11]. At the same time, microbes stabilize SOM residues by binding to soil minerals or forming iron or silicon precipitates, thereby increasing the soil organic carbon (SOC) pool [12]. SOC is a critical component of soil that contributes to its fertility and health [13]. In particular, in shallow soil layers, the SOC plays an important role in improving the soil granular structure [14] and increasing the soil thickness [15]. In addition, there are interactions between microorganisms and environmental disturbances. Heavy metal pollution, overgrazing, and excessive nitrogen application all decrease the diversity of soil microorganisms and disrupt the community structure [16,17,18]. However, microorganisms are able to function in detoxification and anti-interference conditions to a certain extent [16,19]. Some studies had indicated that microorganisms increase grass productivity [20] and confer greater biotic and abiotic stress tolerance to plants [21,22]. Therefore, maintaining microbial stability is extremely important for alpine grasslands on the Tibetan Plateau. Microbes are influenced by environmental factors and respond to perturbations that weaken disadvantages. Thus, microorganisms reflect soil health as biological components of soil.

Chinese *Cordyceps* (*C. sinensis*) is a traditional Chinese medicinal herb [23,24] and also one of the indicator species of ecological changes on the Tibetan Plateau [25]. Modern pharmacological studies have revealed the potential of *C. sinensis* in the treatment of diseases of the kidney [26], liver [27], nervous system [28] and cardiovascular system [29]. In addition, it has anti-tumor, anti-cancer and anti-viral effects, as well as the ability to modulate the immune system, lower cholesterol levels, and act as an antioxidant [30]. Because of the scarcity and protective purposes of *C. sinensis*, studies have focused on the substitutability of *C. sinensis,* as well as the interrelationships between the occurrence of *C. sinensis* and the environment [31]. Recent studies have examined the metabolic spectrum of related species and genomic variations within Chinese *Cordyceps* [32,33], but no reliable substitute for *C. sinensis* has been identified yet.

Studies have shown that Chinese *Cordyceps* is a complex of larvae of *Hepiaua larva* and fungi *Ophiocordyceps sinensis* [34]. The Tibetan Plateau region is an alpine region with low temperatures, large temperature differences between day and night, abundant precipitation, and high relative humidity [35,36]. These climatic factors ensure the growth and reproduction of Chinese *Cordyceps* [25]. The abundance of *Astragalus* and *Polygonum viviparum* provides a favorable food source for bat moth larvae. Its special environmental factors and vegetations composition have made most of the Tibetan Plateau an important region for Chinese *Cordyceps* production [25]. The growth of Chinese *Cordyceps* in soil is demanding, and the soil required for growth necessitate appropriate water storage properties and air permeability [37]. Soil’s physicochemical properties and microbial community composition are associated with the occurrence of Chinese *Cordyceps* [26,34,38]. Limited studies have reported that soil pH, C, N, and K can affect the occurrence of *Cordyceps* [31,39]. The NH_4_^+^-N/NO_3_^−^-N and the moisture of the soil are important factors in the construction of a soil microbial community favorable to the growth of *Cordyceps*. A low level of bacterial and fungal diversity can be suitable for the occurrence of Chinese *Cordyceps* [31,40]. Thus, the instability of soil physicochemical properties and microbial communities affects the regeneration and reproduction of Chinese *Cordyceps*. In order to collect more cordyceps, the soil and vegetation of *Cordyceps* origin are over-trampled and excessively excavated. Over-collection is one of the factors affecting the alteration of soil physicochemical properties and microbial communities in *Cordyceps* production areas [25,41].

Wild *Cordyceps* is an important source of income for herders on the Tibetan Plateau [41]. With the gradual increase in market demand [42], the environmental degradation due to Chinese *Cordyceps* collection is worsening. *Cordyceps* are very difficult to collect and require frequent tramping and the excavation of grassland topsoil and vegetation. Excessive human trampling and disturbance can lead to soil compaction and vegetation degradation and have implications for ecological problems such as the accumulation of soil total nitrogen and organic carbon, as well as grassland degradation [43]. To keep the Chinese *Cordyceps* as intact as possible to obtain greater profits, herders usually dig deeper when excavating, leaving bare pits and neglecting to fill them in. These potholes can become rodent habitats and cause further damage to grassland. It has been shown that the aboveground biomass of Tibetan alpine grassland is very sensitive to the collection of traditional medicinal herbs [44]. Moreover, the collection of Chinese *Cordyceps* and degradation of vegetation has a negative effect on the reproduction of Chinese *Cordyceps* [41]. However, whether soil microbial diversity and functions change due to collection-related disturbance has not been reported. 

The aim of this study was to determine the variations in soil physicochemical properties and microorganisms across different regions of the Tibetan Plateau. Additionally, we aimed to assess the impact of the economic activity of Chinese *Cordyceps* collection on soil microbial communities. To achieve this goal, we selected five distinct regions (Zaduo (ZD), Yushu (YS), Maqin (MQ), Henan (HN) and Hualong (HL)) within Qinghai Province for the study. These were the important *Cordyceps* production areas [25]. Each region was categorized into three zones: collection and non-collection areas of producing and non-producing areas with regard to Chinese *Cordyceps*. Soil samples were collected from these zones. The selected physicochemical properties and enzymatic activities of the soil were measured and the microbial communities were metagenomically sequenced. In this research, (1) we assessed differences in soil nutrient properties and associated enzyme activities in five different regions of the Qinghai Province, and the collection and non-collection areas of producing and non-producing areas with regard to Chinese *Cordyceps*. Although soil nutrient variations between collecting and non-collecting areas were not significant [38], there were large variations between producing and non-producing soils, which might provide rich organic matter for the growth of *Cordyceps sinensis* (medicinal fungi) [24,38,40]. Then, (2) we analyzed the effects of Chinese *Cordyceps* collection on soil microbial diversity, community structure, and functioning in different production area environments using metagenomes. Bacteria are the largest proportion of microbial communities in the soil of the *Cordyceps* production area, with distinct memberships compared to non-production areas. Collecting reduces the tolerance of microorganisms in extreme environments, with greater energy and material consumption required to acquire ecological niches [45]. Finally, (3) we analyzed the interactions between soil microbial communities and environmental factors. Long-term harvesting activities accelerate the decomposition of organic matter by altering microorganisms, thus reducing soil nutrients.

## 2. Results

### 2.1. Soil Nutrients and Enzyme Activity

#### 2.1.1. Soil Nutrients 

**Figure 1:**
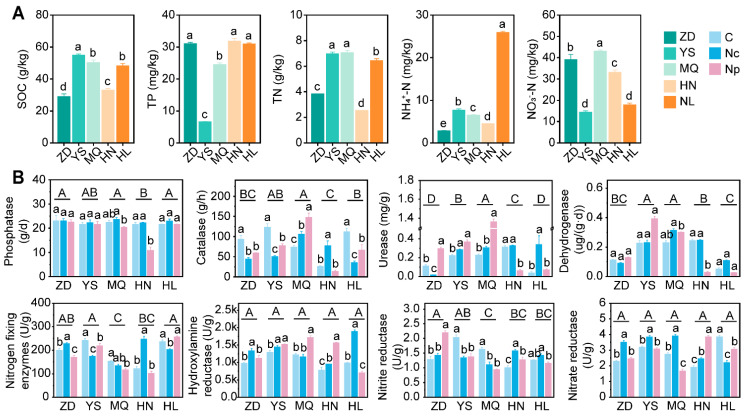
(**A**) Soil physicochemical properties and (**B**) several enzyme activities in the C, Nc and Np of the five regions. Collecting areas of Chinese Cordyceps-producing areas (C); non-collecting areas of Chinese *Cordyceps*-producing areas (Nc); Chinese *Cordyceps* non-producing areas (Np). Zaduo (ZD), Yushu (YS), Maqin (MQ), Henan (HN)n, and Hualong (HL) in Qinghai Province. Different lowercase letters denote significance differences (*p* < 0.05) between the C, Nc and Np, and uppercase letters denote significance differences (*p* < 0.05) between the origins of Chinese *Cordyceps* in Qinghai Province.

The observed variability in soil nutrient composition across the five regions (ZD, YS, MQ, HN, and HL) on the Tibetan Plateau may be attributed to differences in altitude, climatic conditions, and topographic characteristics. Soil samples from these regions exhibited high heterogeneity. Based on the analysis of SOC, P, and N, soil samples from ZD, HN, and MQ were grouped together, while YS and HL formed a separate cluster (Figure S2A). Notably, ZD and HN demonstrated comparable levels of SOC, N, and P (Figure 1A). In the soil samples from the ZD, MQ, and HN regions, nitrogen predominantly existed as nitrate nitrogen. In contrast, the YS region exhibited higher SOC and P content, but lower levels of NH_4_^+^-N and NO_3_^−^-N. The SOC and P characteristics of HL were similar to those of YS, but with a higher concentration of N, primarily in the form of an ammonium ion.

According to our previous study, we found no significant differences in soil SOC, TN, and TP between collection and non-collection areas [38,46,47], but there were significant differences in SOC, TN, and TP contents between production and non-production areas. The SOC content of the soils in the production areas was 86.44–197.13 g/kg, the TN content was 2.71–8.24 g/kg, and the TP content was 0.30–0.56 g/kg [38]. The SOC content of non-producing soils was 28.82–54.81 g/kg, the TN content was 2.52–7.05 g/kg, and the TP content was only 6.51–31.76 mg/kg. The SOC, TN, and TP contents were significantly higher in the producing areas than in the non-producing areas.

#### 2.1.2. Soil Enzyme Activity

We analyzed the enzyme activities in 45 soil samples from both Chinese *Cordyceps*-producing areas (C and Nc) and non-producing areas (Np) across five regions. The collection of Chinese *Cordyceps* significantly influenced several soil enzyme activities (Figure 1B). Due to variations in physicochemical properties, soil enzymes from different regions responded differently to the collection process. Soil catalase, urease, nitrogen-fixing enzymes, dehydrogenase, nitrate reductase, and nitrite reductase were particularly sensitive to collection. In contrast, phosphatase and hydroxylamine reductase activities remained largely unchanged. Specifically, catalase activity was notably higher in the C than in the Nc and Np, increasing by 39.0% and 20.7%, respectively. Urease activity was significantly lower (by 63.86%) in the C than in the NC, but there was no significant difference compared to the NP. In addition, soil urease activity in the NP of MQ was higher than that of other samples. There was consistency in the phosphatase activity of the other samples, except for the significantly low phosphatase activity of the soil in the NP of HN. Significant differences in nitrogen-fixing enzyme, hydroxylamine reductase, nitrate reductase, and nitrite reductase activities were detected among the C, Nc, and Np (Figure 1). The results indicate that there were differences in the way nitrogen-related enzyme activities were affected by the collection of Chinese *Cordyceps* in different soil environments, and also in the size of the impact.

### 2.2. Soil Microbial Diversity and Community Structure

#### 2.2.1. Sequencing Results and Microbial Diversity

**Figure 2:**
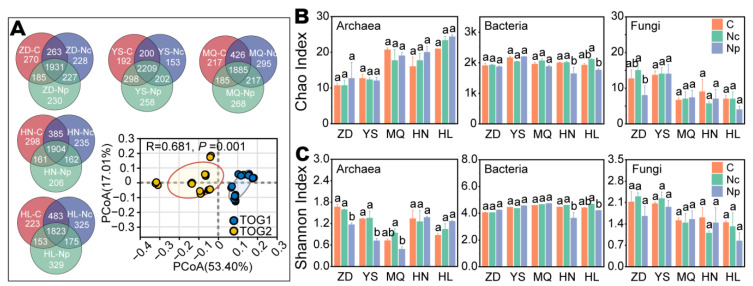
Microbial community α- and β-diversity. Shared and unique microorganisms and PCoA of the soil bacterial community (**A**); Chao1 and Shannon index values of archaea, bacteria, and fungi (**B**,**C**). TOG1 clustering contains all samples from ZD and YS, and TOG2 contains all samples from HL and HN. Collecting areas of Chinese *Cordyceps*-producing areas (C); non-collecting areas of Chinese *Cordyceps*-producing areas (Nc); Chinese *Cordyceps* non-producing areas (Np). Zaduo (ZD), Yushu (YS), Maqin (MQ), Henan (HN), and Hualong (HL) in the Qinghai Province. Different lowercase letters denote significance differences (*p* < 0.05) between C, Nc, and Np.

We obtained an average of 45.82 and 43.77 million raw reads and clean reads, respectively, of 150 bp sequences per sample (Supplemental Data). Only 4.46% of the sequences failed quality control checks. On average, only 10.1% of sequences were successfully annotated (Supplemental Data), consistent with previous metagenomic sequencing studies of diverse microbial habitats [48]. PCoA analyses of soil microbial communities at the species level across different regions indicated that the Chinese *Cordyceps* collection led to a distinct clustering separation among C, Nc, and Np (Figure S1A–E). It suggested that collecting Chinese *Cordyceps* altered the soil microbial composition. Further PCoA performed on regional microbiological samples showed variations, although not all samples were distinctly separated by region (Figure S1F). The microbial community was annotated using the NR database, and a total of 79 phyla (Archaea: 8, Bacteria: 59, Eukaryota: 12) were identified. To explore similarities and differences in microbial communities from various regions, including both producing (C and Nc) and non-producing areas (Np), the unique and shared archaea, bacteria, and eukaryota were analyzed at the species level. The results revealed that few archaebacterial and fungal species were unique to the 45 subgroup samples (Figure S2B). Thus, the effect of collection on the diversity of archaeal and fungal communities was not significant. In ZD and HN, the collection of Chinese *Cordyceps* increased the number of unique soil bacterial species (Figure 2A). The levels of soil-specific bacteria in the YS, MQ, and HL regions were reduced by collection. Although PCoA analyses showed significant variations in microbial communities in the different monitoring areas, this was not reflected in the alpha-diversity (Figure 2B).

We categorized the bacterial microbiota from different regions into two groups: TOG1 (ZD and YS) and TOG2 (HN and HL). The bacterial community structures of these two groups exhibited divergent trends (see Figure 1A). The elevation of the TOG1 test area was lower than that of TOG2. However, soil microbiological samples from TOG2 became more dispersed due to collection activities. Notably, TOG2 was more impacted by the collection of Chinese Cordyceps than TOG1.

#### 2.2.2. Microbial Community Composition and Structure

Bacterial communities accounted for 95.24–99.83% of the microorganisms in the five major producing regions. Archaea and fungi accounted for 0.13–4.73%. We analyzed the microbial community structure at the phylum and the genus levels, respectively. The microbial composition significantly differed among treatments (*p* < 0.05). The dominant phyla of archaea were Thaumarchaeota (92.37%) and Crenarchaeota (6.62%, Figure S3A). The top four dominant archaeal genera could not be classified by genus, but their abundance varied significantly in C, Nc, and Np (Figure S3B). The abundance of Thaumarchaeota increased significantly in the production areas (C and Nc) of ZD and HL (Figure S3A). However, the opposite was true for YS, MQ, and HN. With the exception of MQ and YS, the short-term restricted (Nc) and long-term restricted (Np) collection of Chinese *Cordyceps* reduced the abundance of Crenarchaeota (Figure S3A). Collection significantly altered the relative abundance of Thaumarchaeota and Crenarchaeota, with significant differences in producing and non-producing soils.

**Figure 3:**
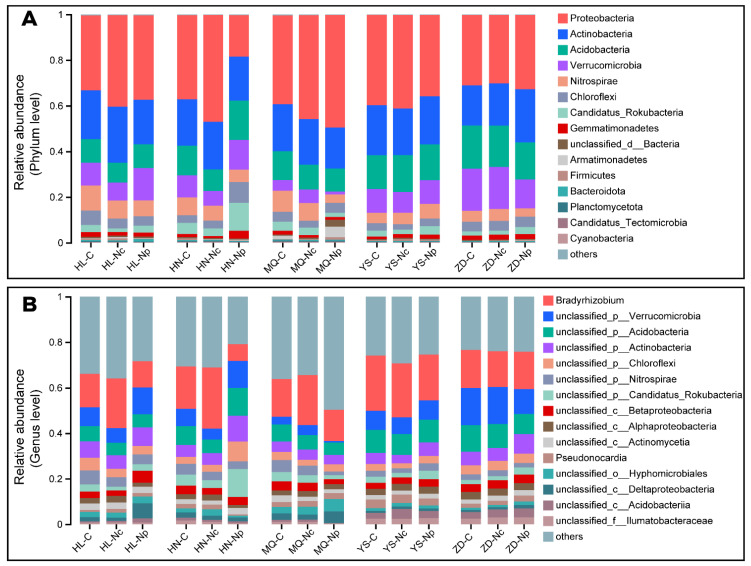
Bacterial community composition and structure at the phylum (**A**) and genus level (**B**). All unidentified microbes in the top 15 relative abundances were categorized as “Unidentified”, and all after 15 relative scores were categorized as “Other”. Collecting areas (C); non-collecting areas (Nc); non-producing areas (Np). Collecting areas of Chinese Cordyceps-producing areas (C); non-collecting areas of Chinese Cordyceps-producing areas (Nc); Chinese Cordyceps non-producing areas (Np). Zaduo (ZD), Yushu (YS), Maqin (MQ), Henan (HN)n, and Hualong (HL) in Qinghai Province.

A total of 8 phyla at the bacterial phylum level had a relative abundance of more than 1% of the readings (Figure 3). Proteobacteria, Actinobacteria, Acidobacteria, and Verrucomicrobia were identified as the dominant microbes in all regions, with relative abundances of 36.17%, 19.13%, 12.89% and 12.88%, respectively. With the exception of ZD, *Proteobacteria* were significantly less abundant in C than in Nc (Figure 3A). In HL and HN, long-term restricted collection (Np) increased the abundance of Verrucomicrobia, but the opposite was true for MQ, YS, and ZD. The relative abundance of Actinobacteria and Acidobacteria did not differ significantly between collection protocols. Less abundant (1.7–7.45%) phyla included Nitrospirae, Chloroflexi, Candidatus_Rokubacteria, and Gemmatimonadetes (Figure 3A). A bare 34.9% of bacteria-related reads were annotated at the genus level (Figure 3B). Collecting Chinese *Cordyceps* altered the abundance of *Bradyrhizobium*. There were significant differences in the abundance of the top five microbial genera in soil when comparing producing and non-producing areas (Figure 3B).

The dominant eukaryotic phyla were Ascomycota (38.93%), Arthropoda (22.09%), Streptophyta (17.46%), and Chordata (13.14%). Less abundant (1.07–5.44%) phyla included Mucoromycota, Basidiomycota, and Chytridiomycota (Figure S4A). There were 18 eukaryotic genera with relative abundances exceeding 1%. The eukaryotic dominant genera were *Allacma* (20.77%), *Aspergillus* (19.03%), *Chiloscyllium* (13.14%), *Glutinoglossum* (88.46%), and *Lupinus* (6.25%, Figure S4B). The relative abundances of Ascomycota and Chordata were higher in C than in Np for HL, HN, and ZD, and the relative abundance of Ascomycota was lower in C than in Np for MQ and YS (Figure S4A). The opposite was true for Arthropoda. 

**Figure 4:**
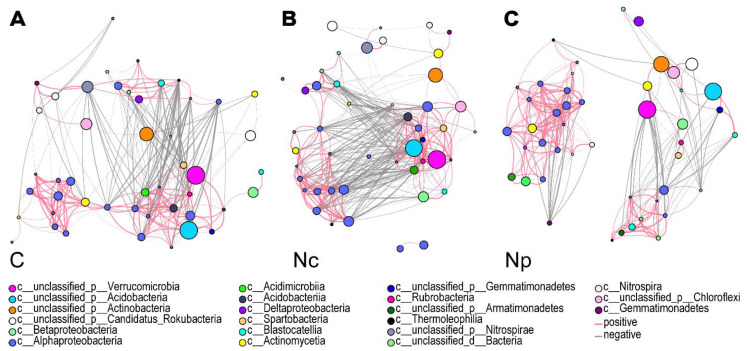
The correlation network analysis of bacterial community (top 50 abundance) in C (**A**), Nc (**B**), and Np (**C**). Each node represents a species, and species of the same class are set to the same color when visualized. The node size in the graph signifies the species abundance, with larger nodes corresponding to greater abundance. Line color signifies correlation: red represents a positive correlation between species, while gray denotes a negative correlation. Line thickness corresponds to the magnitude of the correlation coefficient; a thicker line signifies a stronger correlation between species. The number of lines illustrates the interconnectedness among species, with a higher line count indicating closer connections. Collecting areas of Chinese *Cordyceps*-producing areas (C); non-collecting areas of Chinese *Cordyceps*-producing areas (Nc); Chinese *Cordyceps* non-producing areas (Np).

To investigate the effects of *Cordyceps* collection on the complexity of archaeal, bacterial, and microbial interactions, we constructed correlation networks. The connectivity characteristics of the networks of archaea, bacterial communities, and fungal communities, respectively, changed significantly with the collection activities. At the strong correlation level (|R| > 0.8, *p* < 0.001), soil bacterial communities in Chinese *Cordyceps*-producing areas (C and Nc) were closely related (Figure 4A,B). The average number of nodes per bacteria linkage degree is 26.9, 30.0, and 22.4 for C, Nc, and Np, respectively (Figure 4A, Supplemental Data). However, within the non-producing areas (Np), the bacterial communities formed two clusters, which were loosely associated with each other (Figure 4C). The top three bacterial nodes in terms of abundance were Verrucomicrobia, Acidobacteria, and Actinobacteria. Verrucomicrobia connectivity was 30 in C and 38 in Nc, but only 24 in Np. Among the top 50 bacterial species by abundance, 17–19 nodes belonged to Alphaproteobacteria, which showed positive correlations among species with no significant differences between communities in C, Nc, and Np. Collection altered correlations involving Gemmatimonadetes. In non-producing soils (nodes: 1; degree: 21; clustering: 0.776), Gemmatimoadetes had a strong negative correlation with other nodes. In producing soils, Gemmatimoadetes showed complex correlations in C (nodes: 1; degree: 28; clustering: 0.74), Nc (nodes: 1; degree: 28; clustering: 0.74), and Np (nodes: 1; degree: 1; clustering: 0.74). The average number of nodes per archaea linkage degree is 12.6, 11.9 and 8.9 for C, Nc, and Np, respectively. Nitorsosphaeraceae involved the dominant nodes in the archaeal community. In comparison to Np (nodes: 4; max node degree, MND: 12; max node clustering, MNC: 0.68), Nitorsosphaeraceae holds a more central position in the correlation networks for both C (nodes: 10; MND: 23; MNC: 0.656) and Nc (nodes: 10; MND: 20; MNC: 0.747). The average number of nodes per eukaryote linkage degree is 6.6, 6.3, and 5.3 for C, Nc, and Np. In the eukaryotic network of C soil, key nodes belong to Fabaceae, Thelebolaceae, Cyperaceae, Pseudeurotiaceae, and Helotiaceae. In contrast, Pinaceae dominates the Nc network, while Hemiscylliidae is the primary node in the Np network. Collecting *Cordyceps* enhances the complexity of the archaeal and fungal communities, respectively (Figure S6). Correlation network analyses at the species level revealed an increased tendency for microbial consortia to form synergistic networks in the soils of the Chinese *Cordyceps* production area (C and Nc), suggesting that they enhanced their ability to exchange information and resist disturbance. 

### 2.3. KEGG Functional Analysis

Chinese *Cordyceps* collection caused changes in KEGG pathways, functional modules, and enzymes in the soil microbial communities of C, Nc and Np. A total of 427 KEGG pathways were identified in 45 soil samples. Sixty-eight pathways were significantly different (*p* < 0.05) among the C, Nc and Np. Four microbial functional pathways, two modules, and three enzymes involved in archaea were significantly altered by collecting Chinese *Cordyceps* (Figure S6A). These pathways were nucleotide excision repair, flagellar assembly, propanoate metabolism, and sulfur metabolism. Only one (steroid hormone biosynthesis) of the functional pathways relevant to eukaryotes differed (Figure S5B).

The collection of Chinese *Cordyceps* altered pathways of nucleotide excision repair, flagellar assembly, propanoate metabolism, and sulfur metabolism in archaea. The assimilatory sulfate reduction module (M00176) and assimilatory sulfite reductase (EC 1.8.7.1) were significantly different between production and non-production areas. This result indicated that collection altered the assimilatory sulfite reductase of soil archaea, affecting the microbial metabolism of sulfur.

**Figure 5:**
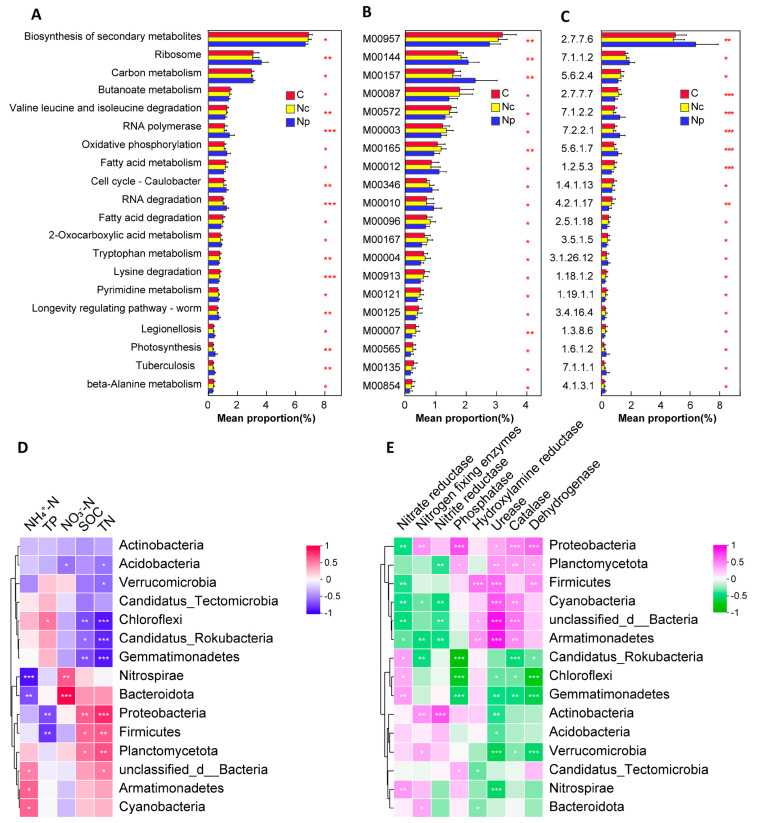
KEGG functional pathways (**A**), modules (**B**), and enzymes (**C**) of bacteria that differentially restrict the collection region. The correlation of enzymes (**D**) and nutrient characteristics (**E**) with the relative abundances of the top fifteen bacterial phyla. Collecting areas of Chinese Cordyceps-producing areas (C); non-collecting areas of Chinese *Cordyceps*-producing areas (Nc); Chinese *Cordyceps* non-producing areas (Np). * significant difference stands at *p* < 0.05; ** significant difference stands at *p* < 0.01; *** significant difference stands at *p* < 0.001.

We screened the bacterial functional pathways that were in the top 20 in terms of relative abundance (Figure 5A). The top 10 functional pathways with significant differences corresponded to bacterial species, with relative abundances exceeding 1%. These pathways were used as the dominant pathways of the analysis in order to illustrate the effects of Chinese *Cordyceps* collection on microbial functions in the ascending biosynthesis of secondary metabolites, ribosomes, carbon metabolism, butanoate metabolism, valine leucine and isoleucine degradation, RNA polymerase, oxidative phosphorylation, fatty acid metabolism, the cell cycle of Caulobacter, and RNA degradation. The dominant pathways were significantly different between the Chinese *Cordyceps*-producing areas (C and Nc) and non-producing areas (Np), but no significant differences were detected in the C and Nc microbial pathways. 

A total of 388 functional modules were pooled to identify microbial functional differences. Seventy-four of these modules were significantly affected by the human disturbance caused by Chinese *Cordyceps* collection (Figure 5B, Table S5). Eight bacterial-related modules had relative abundances of more than 1.00%. Five dominant modules were considered to be significantly upregulated: lysine degradation (M00957), beta-oxidation (M00087), pimeloyl–ACP biosynthesis (M00572), gluconeogenesis (M00003), and the reductive pentose phosphate cycle (M00165). Three dominant modules were considered to be significantly downregulated: quinone oxidoreductase (M00144), F-type ATPase (M00157), and the glyoxylate cycle (M00012). 

To further analyze the metabolic differences among the three soil samples, we classified the 2977 enzymes involved in the samples according to the classification system of the International Commission on Enzymes (Figures S6 and S7): oxidoreductases, transferases, Hydrolases, lyases, isomerases, ligases, and translocases. This result showed that there were no significant differences in the relative abundance of each type of enzyme among C, Nc, and Np (*p* > 0.05). The relative abundance of six significantly different enzyme metabolism-related genes exceeded 1% (Figure 5, Table S6). The synthesis of these enzymes mainly involves DNA repair (2.7.7.6, 2.7.7.7) and ATP synthetic hydrolysis (7.1.1.2, 5.6.2.4, 7.1.2.2, 7.2.2.1).

### 2.4. Relationships Between Microbial Communities and Environmental Factors

To illustrate the relationships between soil chemical parameters and microbial communities in different areas, Pearson correlation analyses were conducted for the top 15 phyla in terms of abundance. The results showed that Acidobacteria, Verrucomicrobia, Chloroflexi, Candidatus_Rokubacteria, and Gemmatimonadetes showed negative correlations with TN, NO_3_^—^N, and SOC (Figure 5D). However, Nitrospirae, Bacteroidota, Proteobacteria, Firmicutes, and Planctomycetota showed positive correlations with these environmental factors. Eight enzymes were also strongly correlated with the top fifteen bacterial phyla in terms of abundance (Figure 5E). The KEGG function in the first fifteen enzymes was strongly correlated with SOC, TP, and TN and weakly with NO_3_^-^-N and NH_4_^+^-N (Figure S8B). With the exception of nitrate reductase, which showed a negative correlation with fifteen major functional pathways, all enzymes were positively correlated with major functions (Figure S8A).

## 3. Discussion

### 3.1. Collection of Chinese Cordyceps Altered Soil Nutrients and Enzyme Activity

In this study, we observed that all regions exhibited elevated SOC contents [35], which could be attributed to the distinctive climatic conditions and vegetation characteristics of the Tibetan Plateau. The abundant SOC facilitates the development of a loose and porous soil structure, thereby supporting soil fertility. The enrichment of C, N, and P in the soil can encourage a bacterial community to predominate, sustaining the diversity of the microbial community [45]. Previous studies have indicated that the collection of *Cordyceps sinensis* influenced certain soil physicochemical properties [38]. Soil C, N, and P contents were significantly higher in producing areas than in non-producing areas. Zhang et al. (2017) [46] conducted the measurement of soil physicochemical properties at varying depths pre- and post-collection of Chinese *Cordyceps*, observing that the soil became weakly acidic and experienced calcium deficiency following excavation, conditions that impeded organic matter decomposition and nitrogen fixation. Liu et al. (2022) [47] documented a significant decline in TN content in soil in their study on the impacts of varied trampling intensities on grasslands. 

Soil enzyme activities from different provenances were significantly altered under collection conditions, but this trend varied from region to region. Soil catalase functions to decompose peroxides, facilitate redox reactions within the soil matrix, and enhance both soil permeability and water utilization efficiency [49]. This enzyme not only augments the fertility of soil but also elevates the count of aerobic microorganisms present [50]. Soil urease activity exhibited a positive correlation with the number of soil microorganisms, the organic matter content, and the amount of quickly acting phosphorus [51,52]. Soil phosphatase plays a pivotal role in the soil phosphorus cycle and the supply of plant phosphorus, and its activity serves as an indicator of soil fertility status and phosphorus availability. However, the collection of Chinese *Cordyceps* has been shown to diminish the efficiency of nitrogen and phosphorus utilization by both soil microbiota and vegetation. Dehydrogenase is a crucial biocatalyst for microbial synthesis and metabolism. It is exclusively located within the cell and provides an accurate reflection of the active state of microorganisms [50]. This enzyme facilitates the conversion of organic materials into inorganic compounds, with the liberated energy sufficing to support microbial growth and reproduction. Furthermore, dehydrogenase offers insights into the soil’s oxidation–reduction status.

The variability in enzyme activities observed across soil samples from different regions might be ascribed to differences in the divergent climatic conditions and vegetation trait characteristics of each region. Denitrification predominantly occurs in the rhizosphere, where fluctuations in soil moisture are pronounced, and in the creation of anaerobic environments that modulate the activity of nitrate reductase and nitrite reductase. Soil possesses the capacity to fix atmospheric nitrogen in the form of ammoniacal nitrogen, thereby enhancing plant nitrogen uptake and growth. Nitrogen-fixing enzymes not only serve as indicators of the soil’s nitrogen fixation potential but also as gauges of ecosystem health. According to Cao et al. (2022) [53], the variations in enzyme activity were primarily due to alterations in microbial biomass and abundance, providing insights into the metabolic differences and functional shifts within microbial communities during collection. The extracellular enzymes secreted by soil microorganisms are pivotal for plant nutrient acquisition [54,55]. This research suggests that the collection of Chinese Cordyceps impacts microbial enzymatic metabolism, consequently influencing plants’ absorption and utilization of soil nutrients. 

### 3.2. Chinese Cordyceps Collection Alters Microbial Composition

Thaumarchaeota contain a complete carbon sequestration pathway and have been shown to be among the most efficient carbon sequestration pathways [12,14]. Collection affects the number of Thaumarchaeota, which is detrimental to soil carbon sequestration. Crenarchaeota are a group of thermophilic and acidophilic archaea [12]. Crenarchaeota include anaerobic heterotrophic organisms that can utilize proteins and sugars as well as sulfur-cycling chemolithoautotrophic organisms that typically live in sulfur-rich soil environments [50,56]. Collection leads to significant changes in the abundance of Crenarchaeota, which inevitably affects the metabolism of sulfur in the soil, and the long-term retention of sulfur in the soil leads to the gradual acidification of the soil.

For the fungal community, collection resulted mainly in the Ascomycota and Mu-coromycota communities being altered. *Cordyceps sinensis* is a complex formed by the growth of mycelium from bat moth larvae parasitized by the fungus *Ophiocordyceps sinensis* of the family *Ciaviceps purpurea* [25,29]. This occurs under complex and demanding conditions. Changes in soil fungal communities in production and non-production areas may be among the key factors in the occurrence of *Cordyceps sinensis*.

The soil microbial communities exhibited significant divergence post-collection, yet they formed stable structural assemblages after prolonged periods of anthropogenic disturbance coupled with multiple environmental influences [56]. This observation also indicates that the soil microbial community possesses robust resilience [57] towards collection activities. Chinese *Cordyceps* is covered by aboveground vegetation. Consequently, the process of collecting wild Chinese *Cordyceps* necessitates continual trampling on grassland to locate it. The soil in the habitat where Chinese *Cordyceps* grows is characterized by a high moisture content [39]. Distinct from the trampling effect of grazing, human trampling in grasslands amplifies soil compaction, diminishing soil water content and permeability. An environment with diminished oxygen levels may favor the proliferation of anaerobic bacteria, leading to an augmentation in the relative abundance of such bacteria within the community. 

The region encompassed in this study was enriched with soil carbon, nitrogen, and phosphorus, which facilitated the rapid proliferation of *Proteobacteria*, thereby establishing it as the predominant phylum in terms of relative abundance [58,59]. The diverse array of glycosyl hydrolases produced by Proteobacteria facilitate nitrogen fixation and promote the decomposition and utilization of organic matter [18]. The Actinomycetes phylum is ubiquitously distributed in soil and is capable of secreting enzymes involved in the carbon cycle, thereby enhancing its participation in the decomposition of plant debris and carbon cycling processes [58]. Acidobacteria can modulate soil pH and engage in soil carbon cycling mechanisms [60]. Typically, the relative abundance of Verrucomicrobia in grassland soils is low [18,60,61]. In this study, however, Verrucomicrobia was found to be abundantly present in the soil.

The Nitrospirae phylum plays an instrumental role in the decomposition of soil mineral nitrogen, thereby enhancing nutrient bioavailability [45]. The abundance of Nitrospirae was notably higher in the areas producing *Cordyceps* compared to the non-producing areas, suggesting that the excavation of *Cordyceps* fosters a reciprocal symbiosis between soil bacteria and plants. Acidobacteria and Chloroflexi phylum were identified as the characteristic and predominantly active flora for ecological restoration, with vegetation capable of instigating alterations in both flora [58]. Chloroflexi possesses a variety of nutritional options, namely, the ability to break down organic matter and the ability to use light energy to fix carbon dioxide [62]. Chinese *Cordyceps* collection significantly reduced the relative abundance of *Bradyrhizobium*, thus affecting nitrogen uptake and utilization by plants. 

### 3.3. Chinese Cordyceps Collection Affects Microbial Community Functions

The nucleotide excision repair (NER) pathway constitutes a pivotal system for addressing microbial DNA damage [63,64]. Collection perturbs the archaeal populations when subjected to DNA damage, instigated by environmental stressors such as ultraviolet radiation, thereby compromising the survival capacity of the archaea in hostile environments. The assembly of flagella may facilitate the adaptation of archaea to harsh habitats, enabling resource acquisition and conferring ecological niche advantages [65]. Perturbations in propanoate and sulfur metabolic pathways influence the tolerance thresholds of archaeal communities to environmental stresses, including soil contamination events.

Collection activities amplify the biosynthesis of soil bacterial secondary metabolites in Chinese *Cordyceps* production areas. Bacteria engage in either antagonistic or reciprocal interactions with other species, mediated through an extensive array of secondary metabolites, thereby enhancing their viability in intricate and intensely competitive ecosystems [66]. These secondary metabolites encompass antibiotics, hormones, and alkaloids [66]. These become enriched, stimulating soil bacteria and making them modulate their responses to environmental shifts. Collecting Chinese *Cordyceps* influences the synthesis of bacterial RNA polymerase and ribosome, subsequently impacting the protein synthesis pathway [67]. The collection of Chinese *Cordyceps* limits the carbon metabolism processes involving microorganisms, mainly the decomposition of SOC and carbon sequestration [59]. In the context of the Tibetan Plateau, the decomposition of organic materials assumes paramount importance, ensuring a sustained supply of accessible carbon for plants and alleviating the ground pressure induced by excessive vegetal detritus. Bacterial secondary metabolite synthesis competes with primary metabolism for amino acid precursors, depleting the amino acid pool [66]. The inhibition of amino acid biosynthesis pathways prevents replenishment, hindering bacterial growth. Carbon, as a fundamental structural component, is crucial in organic synthesis and turnover [4,12]. The disruption of amino acid and carbon metabolism, coupled with heightened secondary metabolism due to *Cordyceps* collection, indicates that bacterial communities must allocate more energy and resources to adapt to environmental changes.

The collection of *Cordyceps* alters the physicochemical attributes of the soil, influencing the relative abundance of diverse bacterial flora, which in turn modulates the functional profile of the microbial community [58]. Microbial organisms promote plant growth and contribute to disease resistance. The microbe–plant interactions directly impinge upon the functioning of grassland ecosystems [21,58]. Bacterial communities are important components of soil ecosystems and participate in the cycling of soil C, N, and P [12,17,35]. The bacterial community is a key component of soil ecosystems. On the one hand, collection led to changes in the bacterial community and impeded bacterial metabolism. On the other hand, collection enhanced the synthesis pathway of secondary metabolites of the bacterial community, and excessive secondary metabolites increased the presence of organic toxic substances in the soil, accelerating the depletion and loss of soil nutrients [35,39,44]. Due to the fragility of the ecosystem of the Tibetan plateau, this mechanism is amplified in a way that affects soil and vegetation health, which in turn affects ecosystem functioning aboveground and in the soil. 

Collection upregulates bacterial lysine catabolism and pimeloyl–ACP modules. Proteobacteria harness lysine catabolism to synthesize the biotin precursor pimeloyl–ACP [68], consequently producing biotin. Biotin is indispensable for all living organisms, playing a cardinal role in fatty acid biosynthesis, amino acid metabolism, and carboxylation reactions during carbohydrate metabolism [69]. The reductive pentose phosphate cycle (Calvin cycle) is enhanced, leading to autotrophic microorganisms displaying a greater abundance in the soil [70]. Glucose synthesis is more favorable for the growth and reproduction of bacterial communities. Quinone oxidoreductase (QOR) performs detoxification, but the associated module is inhibited after collection [71]. At the same time, acquisition leads to the blockage of the bacterial ATPase and Glyoxylate cycle, and energy uptake and metabolism are affected.

### 3.4. Microorganisms Alter Soil Nutrients and Enzyme Activities

The long-term maintenance of diverse soil nutrient profiles, such as SOC, TP, TN, NO_3_^−^-N and NH_4_^+^-N, facilitates the emergence of distinct and stable microbial communities in Chinese *Cordyceps* production areas. Microbial communities in different habitats are distinguished by differences in SOC, N, and P. Soil organic matter can increase the size of soil particles, thus providing different redox conditions to the soil [72]. Microorganisms play an important role in maintaining soil respiration and carbon balance [73]. According to the relevance analysis, the variation in microbial communities under Chinese *Cordyceps* collection conditions is correlated with the activities of enzymes (catalase, urease, dehydrogenase, phosphatase, hydroxylamine reductase, nitrogen-fixing enzymes, nitrate reductase, and nitrite reductase). This reveals that changes in microbial community composition lead to changes in microbial community function. In particular, variation in bacterial phyla is strongly associated with specific enzyme activities [74]. Soil enzymes are products of microbial metabolism and are capable of participating in the biological macrocycle (C, N, P cycle) in the soil, as well as altering the physicochemical properties of the soil. 

### 3.5. Patterns of Microbiological Effects of Cordyceps Collection

The soil nutrient profiles vary across different origins, yet soils from all regions are rich in C, N, and P (Figure 1 and Figure S2A). Anthropogenic activities such as excavation, trampling, and the deposition of domestic waste during collection alter the soil’s physicochemical properties. These changes predominantly influence the beta-diversity, community structure, and functional attributes of archaeal and bacterial taxa (Figures S1 and S3). Thaumarchaeota and Crenarchaeota were identified as the dominant archaeal phyla in the study area. Chinese *Cordyceps* collection affected DNA repair, flagellar assembly, and propionate and sulfur metabolism in archaea (Figure S6A). 

The nutrient-rich soil provides conducive conditions for microbial colonization, resulting in a bacterial community structure dominated by Proteobacteria, Actinobacteria, Acidobacteria, and Verrucomicrobia (Figure 3A). However, the microbial α-diversity indicated that the microbial community did not change significantly due to the collection activities (Figure 2B,C). However, PCoA analysis revealed that collection led to significant changes in the β-diversity of soil bacteria in both producing and non-producing areas. Proteobacteria, Actinobacteria, Acidobacteria, Verrucomicrobia, Nitrospirae, and Chloroflexi were significantly affected by collection (Figure 3A). These bacterial phyla play roles in soil humus decomposition; C, N, and P cycling; and the maintenance of the relative stability of soil pH. At the genus level, a decrease in the abundance of *Bradyrhizobium* was observed, impacting the uptake and utilization of nitrogen by plants (Figure 3B). Correlation networks at the microbial species level indicated that the collection of Chinese *Cordyceps* induced more complex interactions and associations among microorganisms (Figure 4 and Figure S5). 

Metagenomic analyses reveal the impact of changes in microbial abundance on community functions. Pathways associated with RNA polymerase and carbon metabolism were downregulated in the bacterial community (Figure 5). Functional pathways related to bacterial secondary metabolite synthesis (antibiotics, hormones, enzymes) were upregulated, indicating that the bacterial community collectively responded to the adverse effects of collection. The correlation analysis between environmental factors and microorganisms revealed a strong association between them. Meanwhile, enzyme associations with bacterial secondary metabolic pathways were consistent with changes in soil enzyme activities. In summary, the collection of cordyceps perturbs the intrinsic physicochemical properties and microbial community composition of the soil, thereby modifying microbial functions, especially bacterial secondary metabolism. The production of secondary metabolites, in turn, affects soil homeostasis. 

The microorganisms contained in soil samples are extremely rich and diverse. We obtained 45,816,888 reads from the soil microbial samples through metagenomic analysis. However, due to technical and database limitations, only 10.1% of the metagenomic sequencing reads were identified and annotated. The genes contained in the samples were not fully explored and there still exist a large number of microorganisms whose genes and functions are unknown. It may be possible to identify key species that are indicative of environmental change from samples, but this requires further research. 

## 4. Materials and Methods

### 4.1. Field Sites and Sampling

#### 4.1.1. Field Sites

**Figure 6:**
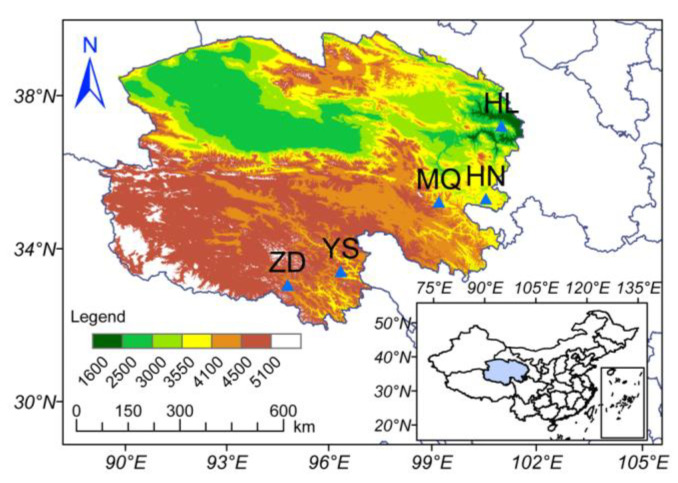
Soil sample collection sites in Qinghai Province. The color gradient corresponds to the elevation information. Zaduo (ZD), Yushu (YS), Maqin (MQ), Henan (HN), and Hualong (HL) in Qinghai Province. These five regions are important *Cordyceps* production areas.

This research was conducted at five representative production areas of Chinese *Cordyceps* in Qinghai, China, including Zaduo (ZD), Yushu (YS), Maqin (MQ), Henan (HN), and Hualong (HL) (Figure 6). These areas were located in the eastern and southern parts of Qinghai Province and had typical plateau continental climates. The average altitude was more than 3700 m in the study area [25]. The average annual temperature remained at −5.6~8.6 °C, and the average precipitation ranges from 15 to 750 mm [19], which made it an alpine region. The vegetation was thin and slow-growing and the major vegetation included *Carex myosuroides* and *Carex tristachya*. Human activities were responsible for the degradation of grasslands in Chinese *Cordyceps*-producing areas, which were more difficult to restore due to climatic constraints. Qinghai Province is considered a biodiversity hotspot and a typical alpine ecosystem [19,25]; therefore, the protection of grasslands in Qinghai Province is particularly important when one takes into account production and benefits, and soil, as an important component of grassland ecosystems, is the focus of this study. 

We identified the distributions of Chinese *Cordyceps* in the grasslands of each area, classified them as producing and (Pr) non-producing areas (Np) on the basis of obtained results, and then divided the producing areas into collecting (C) and non-collecting areas (Nc). The collection of Chinese *Cordyceps* was completely banned in Np; also, the impact of year-round collection has resulted in low Chinese *Cordyceps* resources. The restriction in Nc was for the period of the research. The natural conditions in all production areas are suitable for the growth of Chinese *Cordyceps*. 

#### 4.1.2. Soil Sample Collection

Three randomly selected sites were, respectively, sampled from the C, Nc, and Np in ZD, YS, MQ, HN and HL, respectively. Each soil sample was obtained within the corresponding site following the five-point sampling method. During field sampling, the dominant vegetation in the Chinese *Cordyceps* production region was mainly Carex and tarragon, which had dense roots. Therefore, a mixed soil sample from the 0–15 cm soil layer was selected. The collected mixed soil was passed through a 1 mm standard sieve and immediately transferred to 50 mL centrifuge tubes. The tubes were then placed in liquid nitrogen tanks for temporary storage. After returning to the laboratory, all the soil samples were stored in a −80 °C refrigerator for nutrient, enzyme, and microbial analyses. Subsequently, all soil microbial samples from different production areas were submitted to Majorbio Bio-pharm Technology Co., Ltd. (Shanghai, China) for sequencing. 

### 4.2. Soil Nutrient and Enzyme

#### 4.2.1. Soil Nutrient Measurement

We measured the SOC, TP, TN, NO_3_^−^N, and NH_4_^+^-N contents of the soil samples. SOC was determined using the potassium dichromate oxidative titration method. Two sieved air-dried soil samples of 0.4 g were weighed to measure TP and TN. First, 1.85 g of mixed catalyst (K_2_SO_4_:CuSO_4_:Se = 100:10:1) and 5 mL of concentrated sulfuric acid were added and mixed. The solution was heated (370 °C, 4 h) using an ablator to give a clear pale green color. The ablated solution was added to 20 mL of distilled water to fully dissolve the solution to obtain a TN extract. The other soil sample was mixed with 5 mL of concentrated sulfuric acid and 0.5 mL of perchloric acid. The solution was heated at 280 °C to render the solution transparent and then decocted at 370 °C for 2 h. After cooling, the solution was diluted to 500 mL to measure the TP concentration. The sieved soil samples were weighed to 5.0 g in a 50 mL centrifuge tube, 50 mL of KCl (2 mol/L) extraction solution was added, and the samples were immediately shaken for 1 h and then centrifuged at 8000 rpm for 15 min. The samples were filtered and centrifuged to obtain the samples, and then a SmartChem450 automatic intermittent chemical analyzer was used to measure the soil TP, TN, NO_3_^−^N, and NH_4_^+^-N. All nutrient measurements were performed within one month of sampling. 

#### 4.2.2. Enzyme Activity Assay

The activities of catalase, urease, phosphatase, dehydrogenase, nitrogen-fixing enzymes, hydroxylamine reductase, nitrate reductase, and nitrite reductase were determined according to Guan (1986) [75]. Catalase activity was analyzed by potassium permanganate titration. Phosphatase and urease activities were detected by a colorimetric method using disodium benzene phosphate and sodium phenol–sodium hypochlorite as substrates, respectively. Dehydrogenase, nitrogen-fixing enzymes, hydroxylamine reductase, nitrate reductase, and nitrite reductase activities were determined using the redox method.

### 4.3. DNA Extraction and Metagenomic Sequencing

Soil microbial genomic DNA was extracted using the Fast DNA SPIN Kit (MP Biomedicals, Santa Ana, CA, USA) following the manufacturer’s instructions. The concentration of extracted DNA was measured using a NanoDrop ND-1000 spectrophotometer in order to confirm that all samples reached the sequencing standards (Total DNA yields > 1 μg, concentration > 50 ng/μL, A_260_/A_280_ ratios: 1.8–2.0). After microbial DNA extraction, DNA quality was checked via 1% agarose gel electrophoresis [48]. To minimize operational bias, three replicates of DNA from a single sample were combined. The DNA was fragmented to approximately 350 bp using Covaris M220 to construct a bipartite sequencing library. The metagenomic sequencing of the 45 microbial samples in this study was accomplished to Majorbio Bio-pharm platform (Illumina Nova Seq 6000).

Fastp (https://github.com/OpenGene/fastp, version 0.20.0) was utilized to remove the adaptors and low-quality reads based on a Q score of 20 and a minimum sequence length of 50 bp (Figure S9) [76]. Libraries were normalized to the same size to remove systematic variation between samples. The ORF prediction of contigs from splicing results was performed using Prodigal (https://github.com/hyattpd/Prodigal, version 2.6.3) [48,76]. Genes with nucleic acid lengths greater than or equal to 150 bp were selected and translated into amino acid sequences [77]. The NCBI Genbank database was used for the categorical annotation of metagenomics data, and these data were subsequently annotated to obtain species abundance information using Diamond (https://github.com/bbuchfink/diamond, version 2.0.13) based on the NR (Non-Redundant Protein Sequence Database, 202209) database. The sequences of the non-redundant gene sets were compared with the KEGG (Kyoto Encyclopedia of Genes and Genomes, 202209) database using a desired e-value of 1 × 10^−5^ in Diamond v2.0.13. This data analysis was performed on the Majorbio Cloud Platform (www.majorbio.com, Last used 3 October 2024).

### 4.4. Statistical Analyses

One-way ANOVA was used to determine significant differences between soil nutrients, enzyme activities, and microbial diversity indices among different regions, production areas (Pr: C and Nc), and non-production areas (Np). Principal component analysis (PCoA) based on Bray–Curtis distance was used to elucidate the differences between the microbial communities of samples from different treatments. Mothur (v.1.30.2) was used to analyze Chao1 and Shannon diversity indices for archaea, bacteria, and fungi [45]. Heat maps were created using the R gplot package (v.3.5.3) to compare the top 20 microbial functions with soil nutrient and enzyme activities. Community bar charts and heat maps were visualized using the R language (v.3.3.1) and Vegan (v.2.4.3). Correlation network analysis [45,78] was performed on the 50 most abundant bacterial species in each area, classified by class level, to illustrate relationships between microbial species in the collection and non-collection areas of the production areas and non-production areas. Significant positive (R > 0.80, *p*-value < 0.001) and negative (R < −0.80, *p* < 0.001) correlations [31] were identified and visualized as networks using the Networkx (v.1.11) package for Python (v.3.8.0). This analysis and visualization process was performed on the Majorbio Cloud Platform (www.majorbio.com, Last used 3 October 2024).

## 5. Conclusions

This study indicated that Chinese *Cordycpes* collection had no significant effect on soil’s carbon, nitrogen, and phosphorus content. However, high levels of these nutrients in the production area may favor *Cordyceps* occurrence. Collection significantly altered beta-diversity of archaeal, bacterial, and fungal communities, but did not affect alpha-diversity. Functions related to DNA repair and sulfur metabolism in archaea; carbon metabolism; and amino acid synthesis in bacteria were inhibited, impacting microbial life and resistance. Additionally, collection increased the synthesis of bacterial secondary metabolites associated with soil enzyme activities, accelerating soil organic matter decomposition and hindering nutrient accumulation. The original soil microbial structure was disrupted by collection, impeding the natural occurrence of *Cordyceps*. This study provides microbiological insights for achieving the sustainable and artificial cultivation of *Cordyceps* sinensis production.

## Data Availability

The raw sequencing reads are publicly available under NCBI BioProject under the accession number PRJNA1089979.

## Supplementary Materials

The following supporting information can be downloaded at: https://www.mdpi.com/article/10.3390/ijms252010961/s1.
