# Supplementary material for: Metagenomic Analysis: Alterations of Soil Microbial Community and Function due to the Disturbance of Collecting Cordyceps sinensis
Source: Int J Mol Sci. 2024 Oct 11;25(20):10961. doi: 10.3390/ijms252010961 (PMC11507193; doi:10.3390/ijms252010961)
Supplement: Supplementary file 1 [file ijms-25-10961-s001.zip › ijms-3227601-Supplementary Materials.docx]

**Metagenomic analysis: alterations of soil microbial community and function duo to the disturbance of collecting *Cordyceps sinensis***

**Yangyang Chen^1^, Zhenjiang Chen^1*^, Xiuzhang Li^2^, Kamran Malik^1^, Chunjie Li^1*^**

1 State Key Laboratory of Herbage Improvement and Grassland Agro-ecosystems, Key Laboratory of Grassland Livestock Industry Innovation (Ministry of Agriculture and Rural Affairs), Engineering Research Center of Grassland Industry (Ministry of Education), Gansu Tech Innovation Centre of Western China Grassland Industry, Center for Grassland Microbiome, College of Pastoral Agriculture Science and Technology, Lanzhou University, Lanzhou 730020, China; Mailing address of all authors: No. 222, Tianshui South Road, Chengguan District, Lanzhou City, Gansu Province, China.

2 Qinghai Academy of Animal and Veterinary Science, Qinghai University, Xining 810016, China.

*Correspondence: Chunjie Li 13919861685; chunjie@lzu.edu.cn; Zhenjiang Chen 17358115501; chenzhenjiang@lzu.edu.cn.


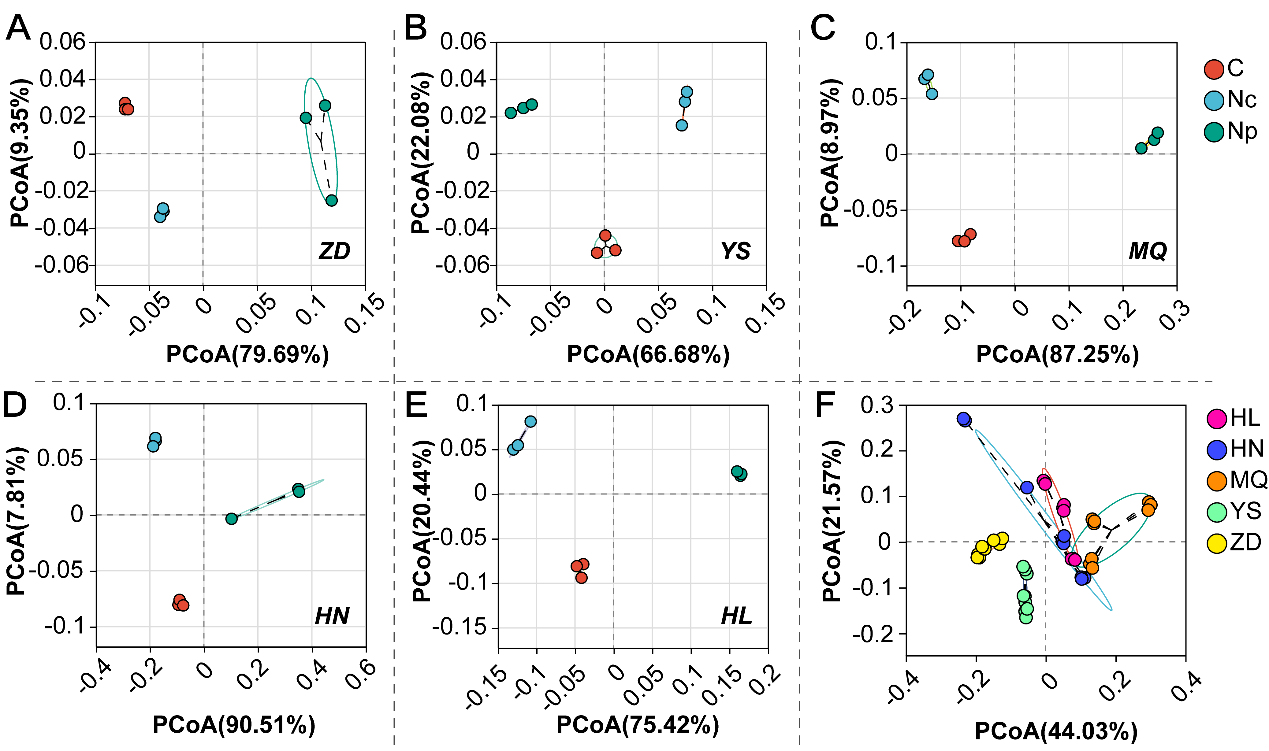


**Figure S1.** PCoA analysis of microbial communities. Microbial community clustering of samples from different regions (A-E). Zaduo (ZD), Yushu (YS), Maqin (MQ), Henan (HN) and Hualong (HL). Microbial community clustering for all samples (F). Collecting areas of Chinese Cordyceps-producing areas (C); non-collecting areas of Chinese Cordyceps-producing areas (Nc); Chinese Cordyceps non-producing areas (Np).


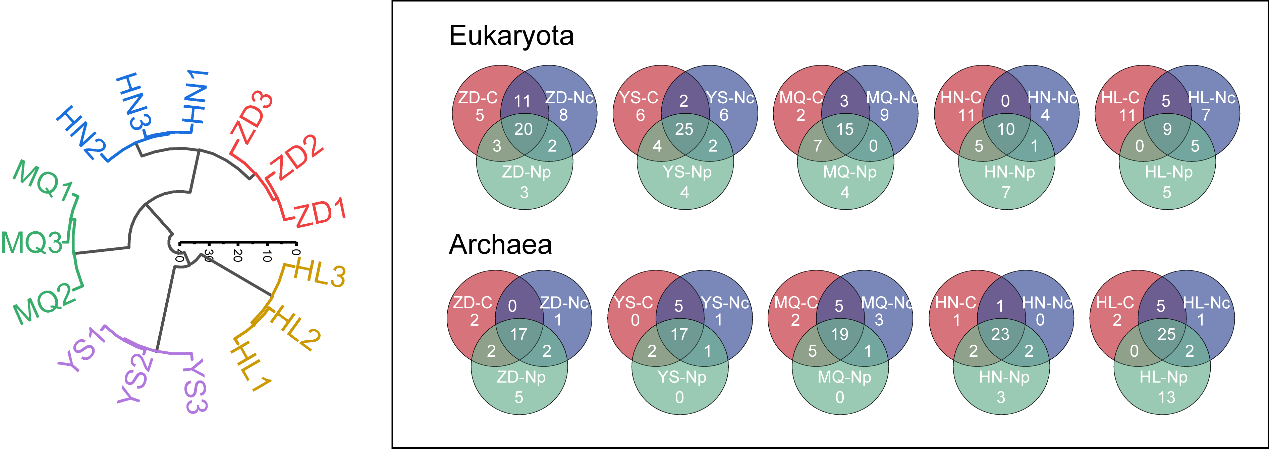


B

A

**Figure S2.** Phylogenetic analysis of physicochemical properties in five regions (A). Veen analyses of eukaryotes and archaea from different Cordyceps producing and non-producing regions (B). Collecting areas of Chinese Cordyceps-producing areas (C); non-collecting areas of Chinese Cordyceps-producing areas (Nc); Chinese Cordyceps non-producing areas (Np).


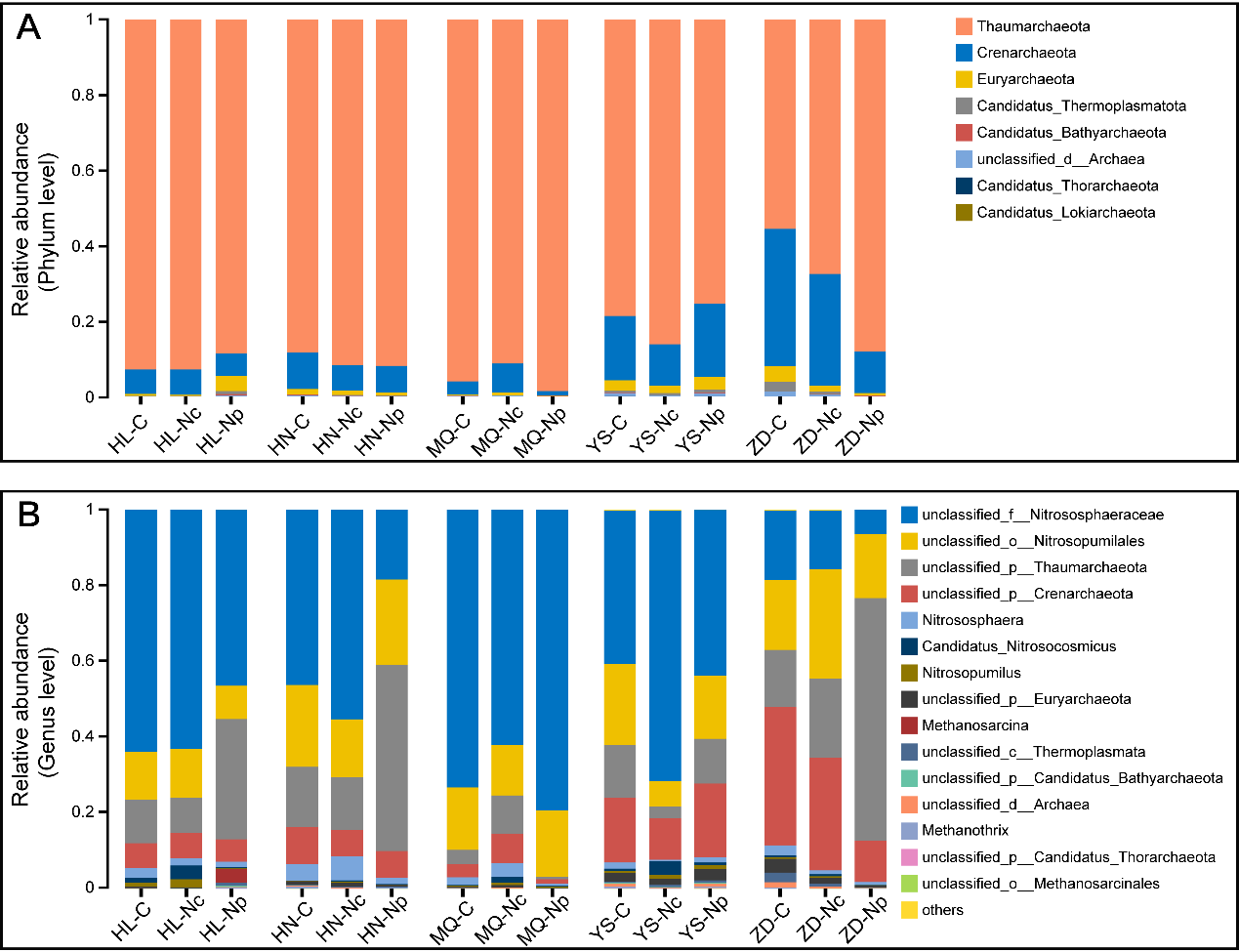


**Figure S3.** Archaeal community structure at the phylum level and genus level. All unidentified microbes in the top 15 relative abundances were categorized as "Unidentified", and all after 15 relative scores were categorized as "Other". Collecting areas of Chinese Cordyceps-producing areas (C); non-collecting areas of Chinese Cordyceps-producing areas (Nc); Chinese Cordyceps non-producing areas (Np).


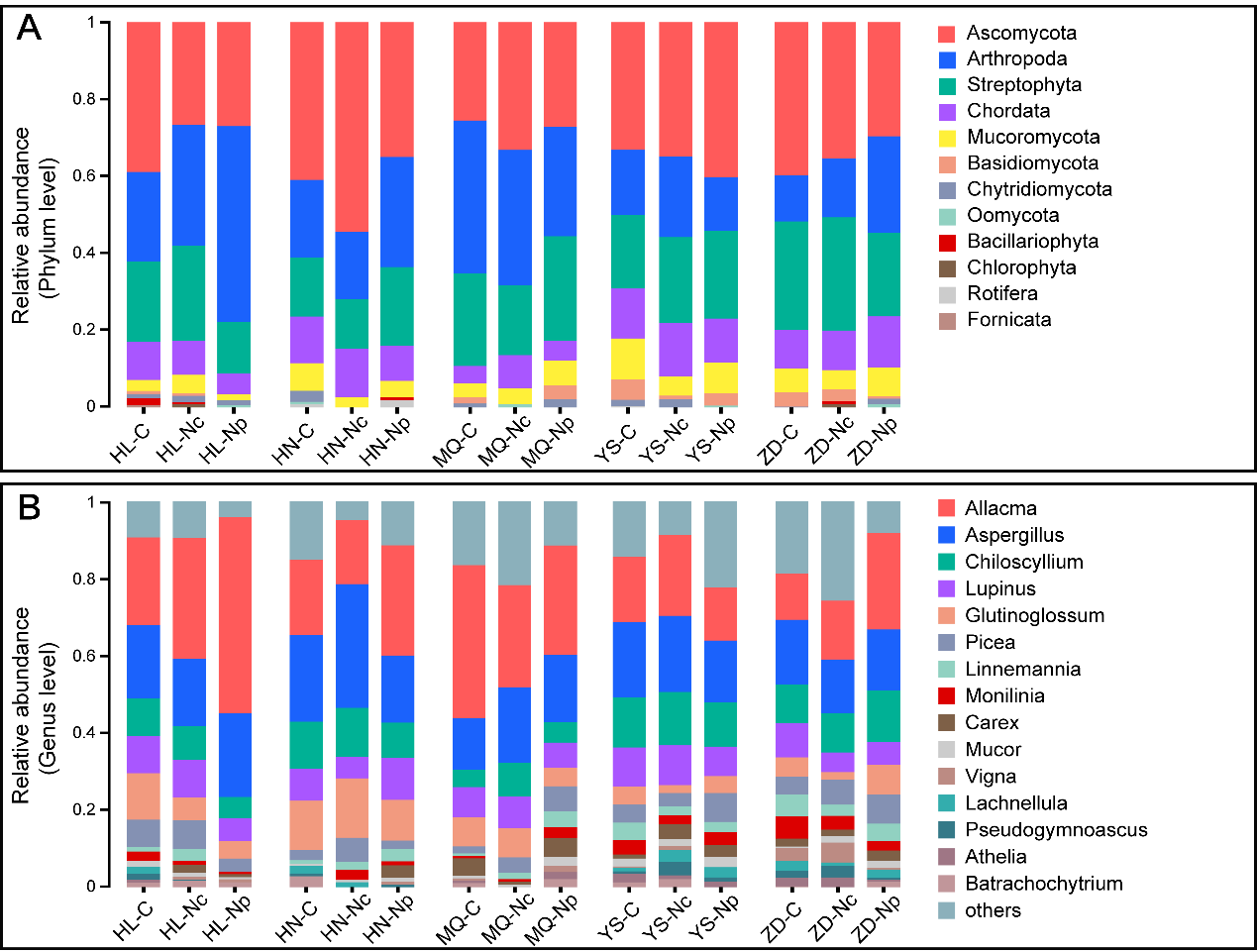


**Figure S4.** Eukaryotic community structure at the phylum and genus levels. All unidentified microbes in the top 15 relative abundances were categorized as "Unidentified", and all after 15 relative scores were categorized as "Other". Collecting areas of Chinese Cordyceps-producing areas (C); non-collecting areas of Chinese Cordyceps-producing areas (Nc); Chinese Cordyceps non-producing areas (Np).


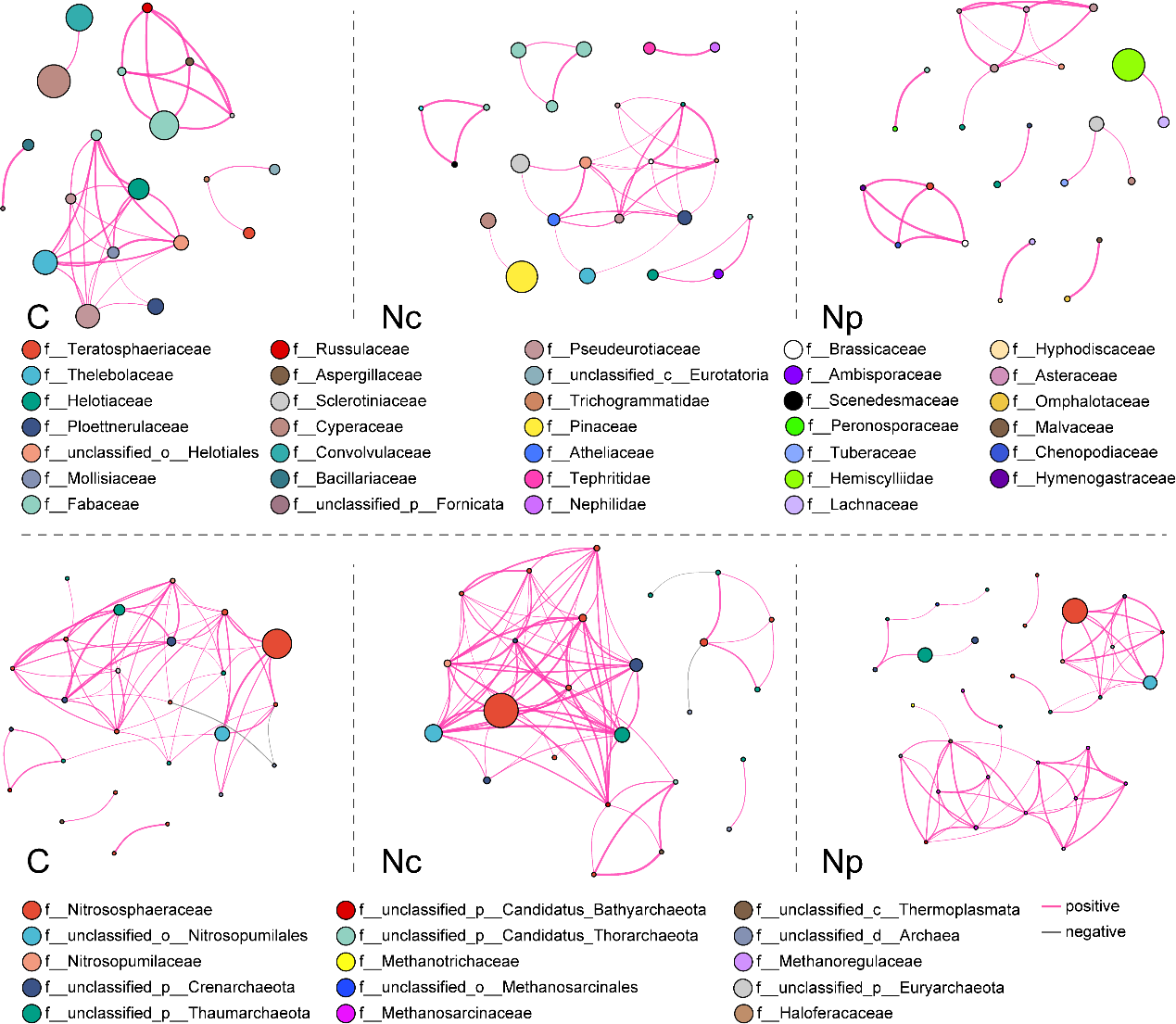


A

B

**Figure S5.** Correlation network analysis of eukaryotic (a) and archaeal (b) community (top 50 abundance) in C, Nc and Np. Node size in the graph signifies the species abundance, with larger nodes corresponding to greater abundance. Line color signifies correlation: red represents a positive correlation between species, while grey denotes a negative correlation. Line thickness corresponds to the magnitude of the correlation coefficient; a thicker line signifies a stronger correlation between species. The number of lines illustrates the interconnectedness among species, with a higher line count indicating closer connections. Collecting areas of Chinese Cordyceps-producing areas (C); non-collecting areas of Chinese Cordyceps-producing areas (Nc); Chinese Cordyceps non-producing areas (Np).


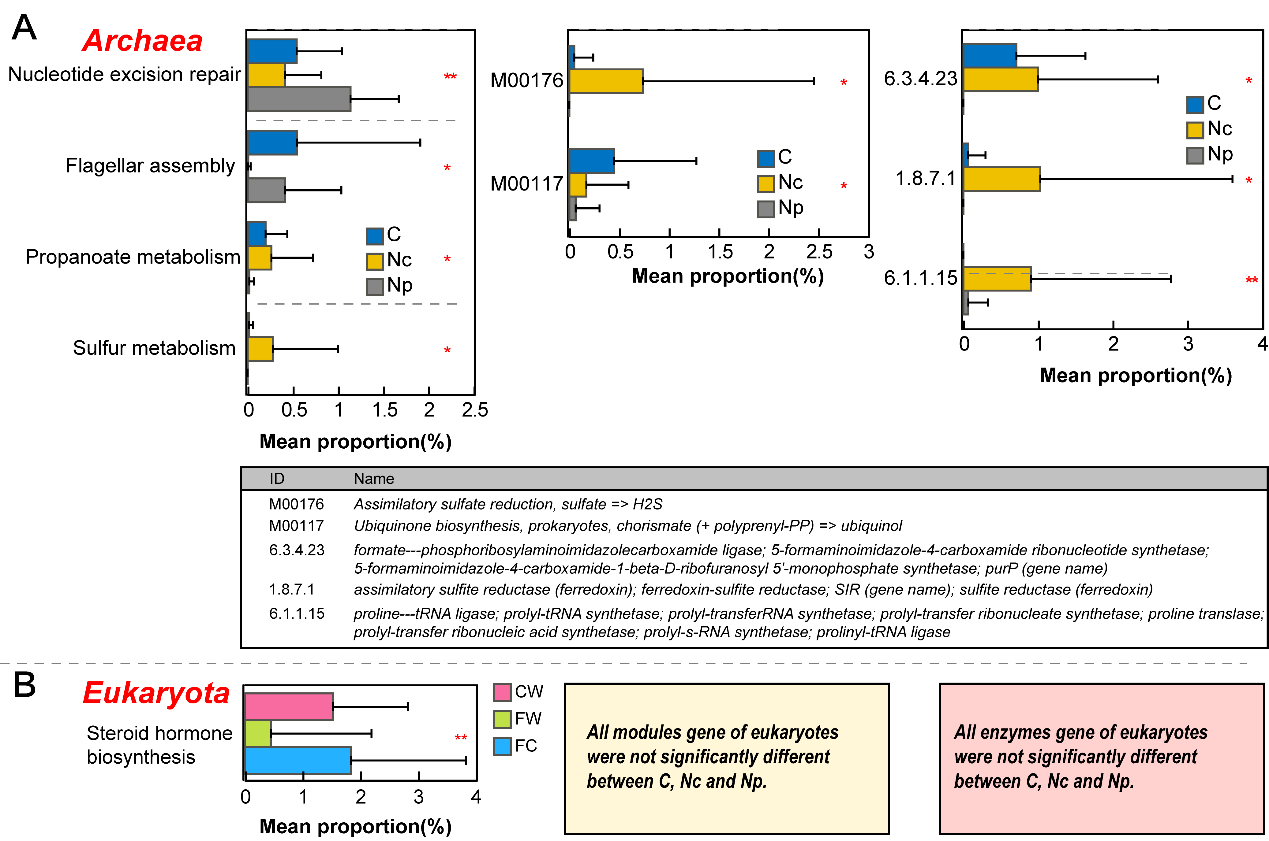


**Figure S6.** Significantly different functional pathways, modules and enzymes associated with archaea and eukaryotes. * Stands significant difference at *P* < 0.05; ** stands at *P* < 0.01; *** stands at *P* < 0.001. Collecting areas of Chinese Cordyceps-producing areas (C); non-collecting areas of Chinese Cordyceps-producing areas (Nc); Chinese Cordyceps non-producing areas (Np).


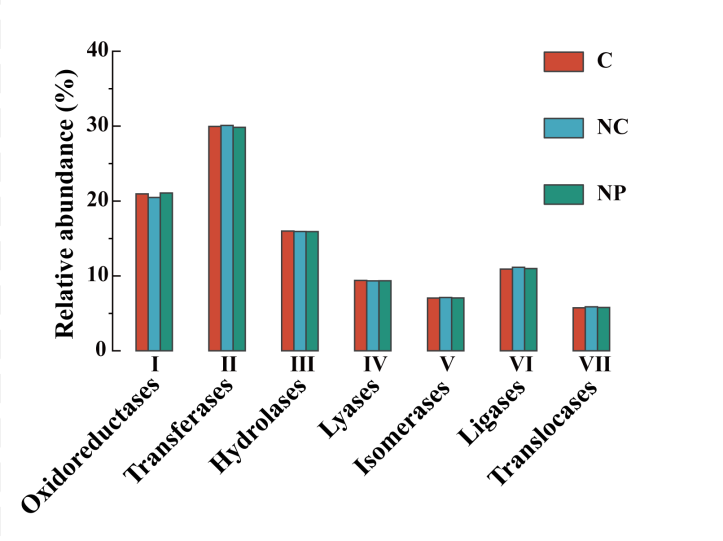


**Figure S7.** Relative abundance levels of different enzymes according to the International Commission on Enzymes classification by KEGG annotation. Collecting areas of Chinese Cordyceps-producing areas (C); non-collecting areas of Chinese Cordyceps-producing areas (Nc); Chinese Cordyceps non-producing areas (Np).


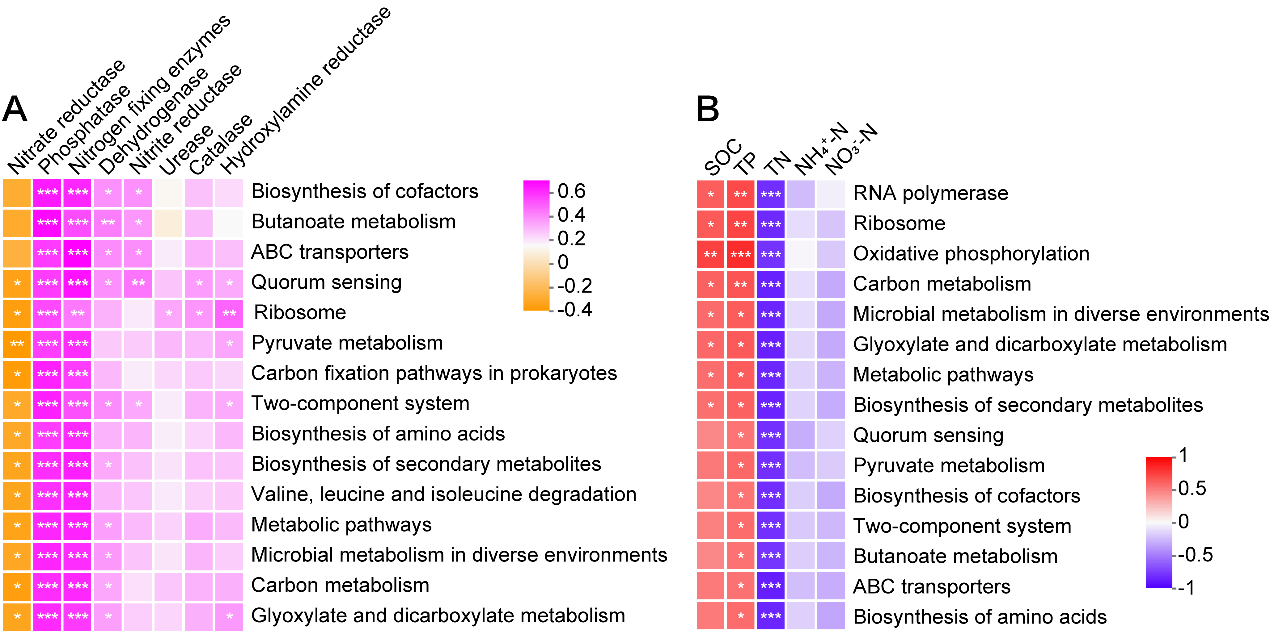


**Figure S8.** Correlation between microbial function and environmental factors in the top fifteen abundances. Collecting areas of Chinese Cordyceps-producing areas (C); non-collecting areas of Chinese Cordyceps-producing areas (Nc); Chinese Cordyceps non-producing areas (Np). * significant difference stands at p < 0.05; ** significant difference stands at p < 0.01; *** significant difference stands at p < 0.001.


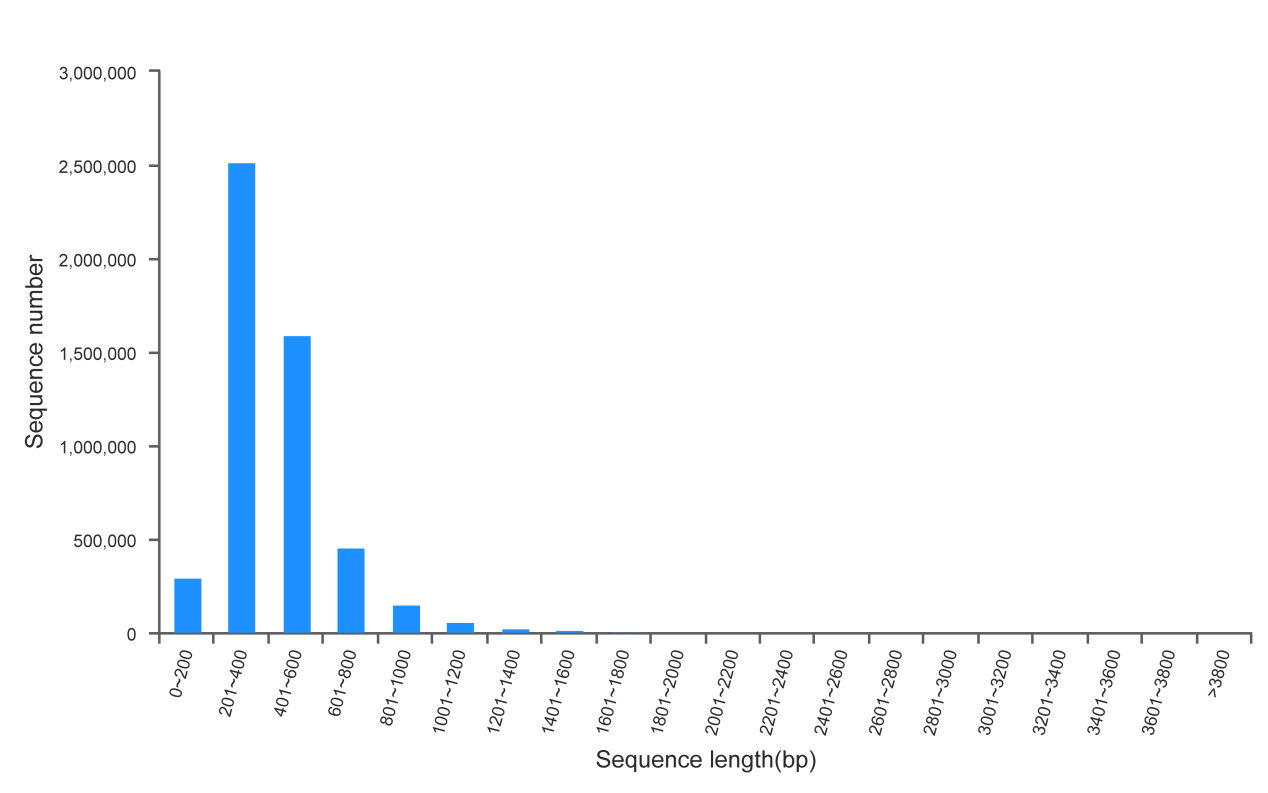


**Figure S9.** Plot of the distribution of non-redundant gene set lengths. The horizontal coordinate is the non-redundant gene set length interval and the vertical coordinate is the number of sequences contained in that interval.

**Table S5.** Definition of dominant KEGG Modules (> 1.0%) in 45 soil samples.

| Module | Definition |
| --- | --- |
| M00957 | Lysine degradation, bacteria, L-lysine => glutarate => succinate/acetyl-CoA |
| M00144 | NADH: quinone oxidoreductase, prokaryotes |
| M00157 | F-type ATPase, prokaryotes and chloroplasts |
| M00087 | beta-Oxidation |
| M00572 | Pimeloyl-ACP biosynthesis, BioC-BioH pathway, malonyl-ACP => pimeloyl-ACP |
| M00003 | Gluconeogenesis, oxaloacetate => fructose-6P |
| M00165 | Reductive pentose phosphate cycle (Calvin cycle) |
| M00012 | Glyoxylate cycle |
| M00346 | Formaldehyde assimilation, serine pathway |
| M00010 | Citrate cycle, first carbon oxidation, oxaloacetate => 2-oxoglutarate |
| M00096 | C5 isoprenoid biosynthesis, non-mevalonate pathway |
| M00167 | Unassigned |
| M00004 | Pentose phosphate pathway (Pentose phosphate cycle) |
| M00913 | Pantothenate biosynthesis, 2-oxoisovalerate/spermine => pantothenate |
| M00121 | Heme biosynthesis, plants and bacteria, glutamate => heme |
| M00125 | Riboflavin biosynthesis, plants and bacteria, GTP => riboflavin/FMN/FAD |
| M00007 | Pentose phosphate pathway, non-oxidative phase, fructose 6P => ribose 5P |
| M00565 | Trehalose biosynthesis, D-glucose 1P => trehalose |
| M00135 | GABA biosynthesis, eukaryotes, putrescine => GABA |
| M00854 | Glycogen biosynthesis, glucose-1P => glycogen/starch |

**Table S6.** Definition of dominant KEGG Enzyme (> 1.0%) in 45 soil samples.

| Enzyme | Sysname |
| --- | --- |
| 2.7.7.6 | nucleoside-triphosphate: RNA nucleotidyl transferase (DNA-directed) |
| 7.1.1.2 | NADH: ubiquinone oxidoreductase |
| 5.6.2.4 | DNA 3'-5' helicase (ATP-hydrolysing) |
| 2.7.7.7 | 2'-deoxyribonucleoside-5'-triphosphate: DNA deoxynucleotidyl transferase (DNA-directed) |
| 7.1.2.2 | ATP phosphohydrolase (two-sector, H+-transporting) |
| 7.2.2.1 | ATP phosphohydrolase (two-sector, Na+-transporting) |
| 5.6.1.7 | ATP phosphohydrolase (polypeptide-unfolding) |
| 1.2.5.3 | carbon-monoxide, water: quinone oxidoreductase |
| 1.4.1.13 | L-glutamate: NADP+ oxidoreductase (transaminating) |
| 4.2.1.17 | (3S)-3-hydroxyacyl-CoA hydro-lyase |
| 2.5.1.18 | RX: glutathione R-transferase |
| 3.5.1.5 | urea amidohydrolase |
| 3.1.26.12 | Endonucleolytic cleavage of single-stranded RNA in A- and U-rich regions |
| 1.18.1.2 | ferredoxin: NADP+ oxidoreductase |
| 1.19.1.1 | flavodoxin: NADP+ oxidoreductase |
| 3.4.16.4 | Preferential cleavage: (Ac)2-L-Lys-D-Ala!D-Ala. Also transpeptidation of peptidyl-alanyl moieties that are N-acyl substituents of D-alanine |
| 1.3.8.6 | glutaryl-CoA: electron-transfer flavoprotein 2,3-oxidoreductase (decarboxylating) |
| 1.6.1.2 | Transferred entry: NAD(P)+ transhydrogenase (Re/Si-specific). Now classified as EC 7.1.1.1, proton-translocating NAD(P)+ transhydrogenase |
| 7.1.1.1 | NADPH: NAD+ oxidoreductase (H+-transporting) |
| 4.1.3.1 | isocitrate glyoxylate-lyase (succinate-forming) |
